# Aflatoxin B1 Impairs Bone Mineralization in Broiler Chickens

**DOI:** 10.3390/toxins16020078

**Published:** 2024-02-02

**Authors:** Deependra Paneru, Milan Kumar Sharma, Hanyi Shi, Jinquan Wang, Woo Kyun Kim

**Affiliations:** Department of Poultry Science, University of Georgia, Athens, GA 30602, USA; dpaneru@uga.edu (D.P.); milan.sharma@uga.edu (M.K.S.); hanyi.shi@uga.edu (H.S.); jq.wang@uga.edu (J.W.)

**Keywords:** aflatoxin B1, broiler bone, bone quality, mycotoxin, Ca and P transporters

## Abstract

Aflatoxin B1 (AFB1), a ubiquitous mycotoxin in corn-based animal feed, particularly in tropical regions, impairs liver function, induces oxidative stress and disrupts cellular pathways, potentially worsening bone health in modern broilers. A 19-day experiment was conducted to investigate the effects of feeding increasing levels of AFB1-contaminated feed (<2, 75–80, 150, 230–260 and 520–560 ppb) on bone mineralization markers in broilers (*n* = 360). While growth performance remained unaffected up to Day 19, significant reductions in tibial bone ash content were observed at levels exceeding 260 ppb. Micro-computed tomography results showed that AFB1 levels at 560 ppb significantly decreased trabecular bone mineral content and density, with a tendency for reduced connectivity density in femur metaphysis. Moreover, AFB1 above 230 ppb reduced the bone volume and tissue volume of the cortical bone of femur. Even at levels above 75 ppb, AFB1 exposure significantly downregulated the jejunal mRNA expressions of the vitamin D receptor and calcium and phosphorus transporters. It can be concluded that AFB1 at levels higher than 230 ppb negatively affects bone health by impairing bone mineralization via disruption of the vitamin D receptor and calcium and phosphorus homeostasis, potentially contributing to bone health issues in broilers.

## 1. Introduction

Aflatoxicosis, a toxic and carcinogenic condition resulting from the ingestion of aflatoxin B1 (AFB1)-contaminated feed, adversely affects poultry growth and health, especially in tropical and subtropical regions where high temperature and humidity promote the growth of fungi of the *Aspergillus* spp. [1]. *Aspergillus* fungi, especially *Aspergillus parasiticus* and *A. flavus*, can colonize crops and grains in the field and during storage and produce AFB1 as a secondary metabolite [2]. AFB1 in the contaminated feed is rapidly absorbed in the duodenum of broiler chickens, then transported via the portal vein into the liver, where it is bio-transformed by cytochrome P450 enzymes into a highly reactive electrophilic metabolite, aflatoxin 8,9-epoxide [3]. As a highly reactive molecule, aflatoxin 8,9-epoxide forms covalent bonds with cellular macromolecules, such as nucleic acids, proteins and phospholipids, and causes various genetic, metabolic, signaling and cell structural alterations [4,5,6,7]. In addition, studies have shown that AFB1 can impair cell function and structure by inducing oxidative stress, which is a major mechanism of AFB1 toxicity [8]. In addition to this, aflatoxicosis affects poultry health by causing intestinal barrier impairment, immune suppression and disruption of enzymes in the liver and other organs [8,9,10,11,12].

Furthermore, modern broiler chickens are genetically selected for fast growth and high meat yield, which imposes a high metabolic demand on their skeletal system to support their growth [13,14,15]. Consequently, rapidly growing broilers exhibit inadequate calcification and increased porosity in their long bones [16]. Bone disorders such as decreased bone-breaking strength caused by aflatoxicosis in broilers have been previously linked to changes in cholecalciferol metabolism [17]. Meanwhile, broilers exposed to AFB1 have shown decreased levels of calcium and phosphorus in their blood serum, which may suggest adverse effects of AFB1 on bone homeostasis [18,19,20]. The liver damage, production of reactive oxygen species and interference with cellular processes caused by aflatoxicosis might intensify bone health issues in modern broilers. Recent human and animal studies have revealed new insights into the bone disorders caused by aflatoxicosis, where AFB1 interferes with the expression and function of the vitamin D receptor (VDR), affecting the metabolism of vitamin D2 and D3 into 25-hydroxycholecalciferol (25-OH) and 1,25-dihydroxycholecalciferol (1,25-OHD), resulting in decreased bone quality [21,22,23].

To comprehensively evaluate the adverse effects of aflatoxicosis on the bone health of broiler chickens, this study employed a novel approach: micro-CT scanning and analyses. This innovative technique assesses three-dimensional bone structure, providing an in-depth understanding of changes in the microstructural and architectural properties of the long bones under increasing doses of AFB1 in the diet. The research aimed to contribute valuable insights into the changes in microstructural and architectural properties of metaphyseal segments of long bones affected by aflatoxicosis, enhancing our understanding of the intricate interplay between AFB1 exposure and bone health in broiler chickens.

## 2. Results

### 2.1. Growth Performance

Aflatoxin B1 inclusion up to 560 ppb did not show significant differences in the growth performance parameters (body weight gain, feed intake and feed conversion ratio) of broilers raised for 19 days (*p* > 0.05; Table 1). No signs of morbidity were observed during the 19-day period. The observed mortality rate during the 19-day period was 1.39, 1.39, 0, 4.17 and 4.17% in the T1 (<2 ppb AFB1), T2 (75–79 ppb AFB1), T3 (150 ppb AFB1), T4 (230–260 ppb AFB1) and T5 (520–560 ppb AFB1) groups, respectively.

### 2.2. Bone Microstructural Changes in Response to Increasing Doses of Aflatoxin B1

**Figure 1:**
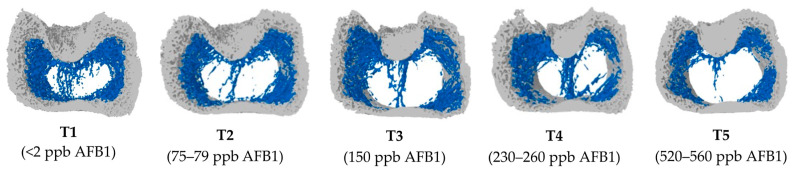
Representative metaphyseal bone structure of femurs on Day 19 after exposure to increasing doses of aflatoxin B1 contaminated diets. The figure depicts cross-sectional images of femoral metaphysis with increasing AFB1 levels from left to right. Each image shows the metaphysis with two distinct regions: outer cortical bone (light grey) and inner trabecular bone (blue). In the femurs from broilers fed diets with higher levels of AFB1 (T4 and T5), there is a clear disruption of the trabecular bone pattern. The blue color network of the trabecular bone appears less dense, with thinner and more fragmented bone strands and spaces between the trabecular bone appear larger. Treatment groups: T1 (<2 ppb AFB1), T2 (75–79 ppb AFB1), T3 (150 ppb AFB1), T4 (230–260 ppb AFB1) and T5 (520–560 ppb AFB1).

On Day 8, no significant differences were observed in the microstructure of the femoral metaphysis across the tested levels of AFB1 (*p* > 0.05; Supplementary Table S1). However, by Day 19, the trabecular bone structure in the metaphysis was notably disrupted, primarily by the T5 group (i.e., 560 ppb) of AFB1 resulting in reduced bone mineral content (BMC; P_Model_ = 0.0331; P_Linear_ = 0.0026) and bone mineral density (BMD: P_Model_ = 0.0214; P_Linear_ = 0.0043), while tending to quadratically reduce connectivity density (Conn.Dn; P_Model_ = 0.0809; P_Quadratic_ = 0.0259; Table 2). Consequently, this led to a decrease in the overall BMC within the total bone segment of the femur metaphysis (P_Model_ = 0.0212; P_Linear_ = 0.0015). The cortical bone structure of the metaphysis exhibited a linear reduction in tissue volume (TV; P_Model_ = 0.0189; P_Linear_ = 0.0002), bone volume (BV; P_Model_ = 0.0429; P_Linear_ = 0.0008), volume of closed pores (Po.V(cl); P_Model_ = 0.0500; P_Linear_ = 0.0060) and volume of open pores (Po.V(op); P_Model_ = 0.0067; P_Linear_ = 0.0004) and porosity percentage (PP; P_Model_ = 0.0337; P_Linear_ = 0.0023) on Day 19. This effect was pronounced for the T4 and T5 groups (i.e., above 230 ppb of AFB1). Consequently, there was a linear decrease in the overall BMC, TV and BV within the total bone segment of the femur metaphysis (P_Model_ < 0.05; P_Linear_ < 0.05). From these results, it is confirmed that AFB1 induced structural changes in the metaphyseal segment of the femur bone when exposed for a longer period (Figure 1). Broilers exposed to the T4 and T5 groups (i.e., above 230 ppb of AFB1) had a disturbed metaphyseal trabecular pattern compared to the T1, T2 and T3 groups (i.e., below 230 ppb of AFB1). These results suggest that AFB1 exposure above 230 ppb for 19 days can potentially impair the trabecular bone, which may compromise the skeletal health and quality of broiler chickens.

### 2.3. Bone Ash Content of Tibia Bone

**Figure 2:**
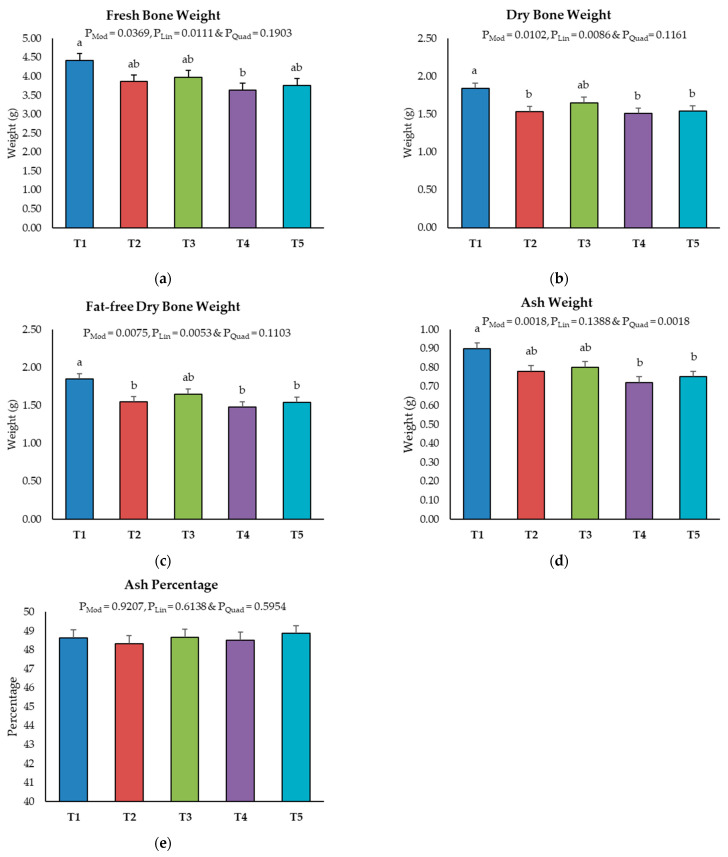
Effect of increasing the dosage of an aflatoxin B1 contaminated diet on tibial bone parameters on Day 19: (**a**) fresh bone weight; (**b**) dry bone weight; (**c**) fat-free dry bone weight; (**d**) ash weight; (**e**) ash percentage. The data relate to the mean values of six tibia bone samples per treatment. The x-axis represents the treatment groups: T1 (<2 ppb AFB1), T2 (75–79 ppb AFB1), T3 (150 ppb AFB1), T4 (230–260 ppb AFB1) and T5 (520–560 ppb AFB1). Statistically significant differences between means are indicated by superscript letters (a, b) above the error bars in each panel.

On Day 8, no significant differences were observed in the bone ash parameters among the different AFB1 levels (*p* > 0.05; Supplementary Table S2). However, by Day 19, a linear reduction in fresh bone weight (FBW), dry bone weight (DBW) and fat-free dry weight (FFDW) were observed with increasing AFB1 levels (P_Model_ < 0.05; P_Linear_ < 0.05; Figure 2a–c). Ash weight (AW) was significantly reduced by AFB1 levels at 230 ppb and above (T3 and T4), compared to the control (T1) (Figure 2d). In contrast, ash percentage was not affected by the tested levels of AFB1 on Day 19 (*p* > 0.05) (Figure 2e). 

### 2.4. Changes in the Expression of Calcium and Phosphorus Transporters

**Figure 3:**
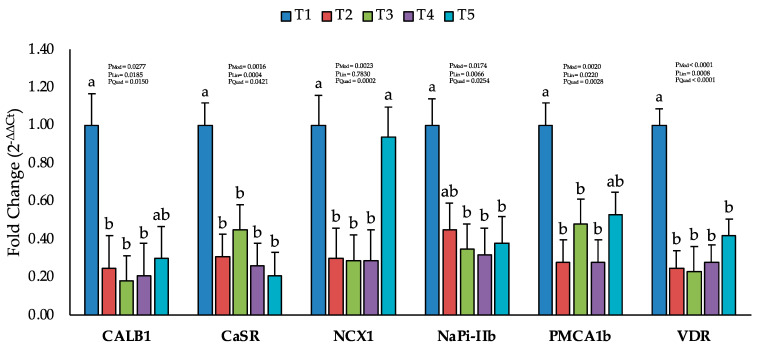
Effect of increasing the dosage of an aflatoxin B1 contaminated diet on mRNA levels of calcium and phosphorus transporter genes on Day 19. The data are presented as the mean values of fold change in mRNA expression of calcium and phosphorus transporter genes using the 2^−ΔΔCt^ method. The data relate to 6 jejunum samples per treatment. The genes are abbreviated as CALB1 for calbindin 1, CaSR for calcium-sensing receptor, NCX1 for sodium-calcium exchanger 1, NaPi-IIb for sodium-phosphate cotransporter IIb, PMCA1b for plasma membrane calcium ATPase 1b and VDR for the vitamin D receptor. Treatment groups: T1 (<2 ppb AFB1), T2 (75–79 ppb AFB1), T3 (150 ppb AFB1), T4 (230–260 ppb AFB1) and T5 (520–560 ppb AFB1). The *p*-values provided for the whole model (P_Mod_), linear regression (P_Lin_) and quadratic regression (P_Quad_). Statistically significant differences between means are indicated by superscript letters (a, b) above the error bars in each panel.

On Day 8, the jejunal mRNA expression of VDR and calcium and phosphorus transporters did not differ from the control group (*p* > 0.05; Supplementary Table S3). However, by Day 19, significant reductions were observed in the mRNA expression of the vitamin D receptor (VDR), calbindin 1 (CALB1), calcium-sensing receptor (CaSR) and type IIb sodium phosphate co-transporter (NaPi-IIb) in the jejunum of broilers with increasing levels of AFB1 (*p* < 0.05; Figure 3). However, the mRNA expression of the sodium-calcium exchanger (NCX1) and plasma membrane calcium ATPase 2 (PMCA1b) were significantly reduced by AFB1 levels up to T3 (230–260 ppb AFB1) but non-significant at T5 (520–560 ppb AFB1), compared to the T1 group (i.e., less than 2 ppb AFB1).

### 2.5. Intestinal Permeability and Tight Junction Proteins

**Figure 4:**
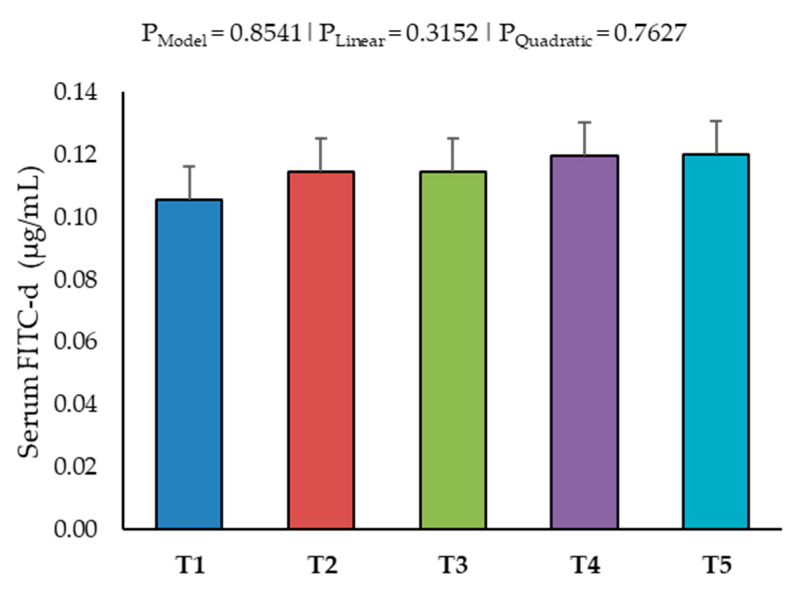
Effect of increasing the dosage of an aflatoxin B1 contaminated diet on the intestinal permeability on Day 19. The data are presented as the mean blood serum concentrations of fluorescein isothiocyanate dextran (FITC-d) in µg/mL for five treatment groups (n = 6/treatment). Treatment groups: T1 (<2 ppb AFB1), T2 (75–79 ppb AFB1), T3 (150 ppb AFB1), T4 (230–260 ppb AFB1) and T5 (520–560 ppb AFB1). The *p*-values provided are: P_Model_ for the whole model, P_Linear_ for linear regression and P_Quadratic_ for quadratic regression.

Intestinal permeability was not affected by AFB1 up to 520–560 ppb on Day 19 (Figure 4).

**Figure 5:**
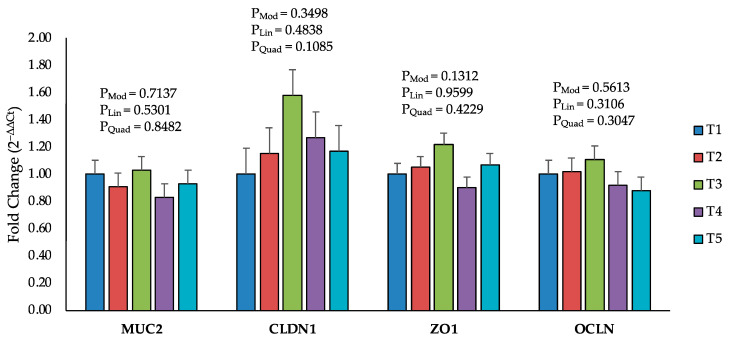
Effect of increasing the dosage of an aflatoxin B1 contaminated diet on mRNA levels of tight junction proteins on Day 19. The data are presented as the mean values of fold change in mRNA expression of tight-junction-related genes using the 2-ΔΔCt method. The data relate to 6 jejunum samples per treatment. The genes are abbreviated as MUC2 for mucin 2, CLDN1 for claudin 1, ZO1 for zonula occludens and OCLN for occludin. Treatment groups: T1 (<2 ppb AFB1), T2 (75–79 ppb AFB1), T3 (150 ppb AFB1), T4 (230–260 ppb AFB1) and T5 (520–560 ppb AFB1). The *p*-values are provided for the whole model (P_Mod_), linear regression (P_Lin_) and quadratic regression (P_Quad_).

The mRNA expression of tight junction proteins was not significantly affected by AFB1 up to 520–560 ppb on Day 8 (Supplementary Table S4) and Day 19 (Figure 5).

## 3. Discussion

Bone disorder is a major challenge for the poultry industry, as it affects the welfare and productivity of the birds [24]. Bone disorders can result from infectious or noninfectious causes, such as bacterial osteomyelitis, viral arthritis, rickets, tibial dyschondroplasia or nutritional factors [25,26]. Birds with bone disorders are often culled, condemned or downgraded at the processing plant, which causes direct economic losses to the poultry producers. Bones serve as essential multi-functional organs, providing structural support, safeguarding the vital organs and functioning as endocrine organs releasing hormones crucial for mineral homeostasis, acid–base balance and serving as reservoirs of energy and minerals [27,28,29]. Despite its pivotal role, the attention to bone health in broiler production has been relatively limited until recently. The high growth rate of modern broilers, leading to significant body weight, has led to the increased susceptibility of these birds to leg weakness and skeletal abnormalities [30]. With AFB1 being a common contaminant in poultry feed in tropical and subtropical regions, the current study used the aflatoxicosis model to explore the potential relationship between bone health and increasing AFB1 exposure. Broilers fed with a low dose of an AFB1 contaminated diet can often maintain normal growth without visible signs of mycotoxicosis, but this does not exclude the possibility of toxic effects on immune function, gut health and liver function, which may lead to bone disorders [31,32].

In the present study, we investigated the effect of increasing doses of AFB1 from <2 ppb to 560 ppb on the bone quality of broiler chickens to determine the threshold dose and duration that induces changes in bone microstructural architectural properties. AFB1 doses up to 560 ppb did not affect the growth performance of broilers raised for 19 days. This is consistent with some previous studies that reported no effects of AFB1 on body weight gain, feed intake and feed conversion ratio of broilers at similar or higher doses administered for a similar duration [7,12,33]. It is possible that a threshold concentration exists, below which AFB1 does not exert a noticeable impact on the physiological processes involved in broiler growth, and the short duration of exposure (19 days) may not have been adequate to manifest growth-related consequences. However, other studies have shown that AFB1 can impair the growth performance of broilers at even lower doses, such as 40 to 500 ppb [34,35,36,37,38]. The discrepancy among these studies may be due to differences in the source and quality of feed ingredients, the presence of other mycotoxins and the nutritional status of the birds. The nutritional status of the birds may affect the absorption, metabolism and excretion of AFB1, as well as the immune response and antioxidant defense against AFB1 toxicity [39,40]. Other mycotoxins in the feed may have synergistic or antagonistic effects on the AFB1 toxicity [7,41]. Therefore, the effects of AFB1 on broiler growth performance may not be linear or dose-dependent but somewhat influenced by multiple factors that modulate the toxicity and metabolism of AFB1.

A significant reduction in tibial ash weight was observed in broilers fed with AFB1 above 230 ppb for 19 days in the current study. Furthermore, the micro-CT analysis revealed impaired trabecular bone structure, as evidenced by the disturbed metaphyseal trabecular pattern and the reduced bone mineral content (BMC) and bone mineral density (BMD) in trabecular bone with AFB1 exposure at 230 ppb or above for 19 days. This study also showed that AFB1 exposure at 230 ppb or above for 19 days reduced the cortical bone structure, as evidenced by the decreased tissue volume (TV), bone volume (BV), tissue surface area (TS) and bone surface area (BS). A metaphyseal segment of the femur bone is the site of active bone remodeling and growth [42], which consists of two types of bone tissue: trabecular and cortical. Trabecular bone is a network of thin plates and rods that provides mechanical support and metabolic functions [43]. Cortical bone is a dense tissue layer surrounding the trabecular bone and provides strength and rigidity [44]. A decrease in the BMC and BMD of trabecular bone with aflatoxicosis indicates the loss of bone strength and metabolic functions [45]. The trabecular bones form a lattice structure, offering a greater surface area for osteoclast attachment, resulting in a higher turnover rate during bone resorption compared to cortical bones [46,47]. The loss in tissue volume and cortical bone volume signifies a reduction in total bone mass associated with aflatoxicosis. In the present study, lower bone mineral content, density and a modified ratio of BV/TV at the metaphyseal trabecular bone may indicate trabecular bone remodeling.

Furthermore, in the present study, the mRNA expression of VDR and calcium and phosphorus transporters were also in line with the decrease in bone mineralization as evidenced by bone ash and micro-CT results. The vitamin D receptor and calcium and phosphorus transporters regulate calcium and phosphorus absorption in the intestine, which are essential for bone mineralization and quality [48]. A decrease in the mRNA expression of these genes implies a decrease in the protein expression and activity of these transporters, which may compromise the intestinal uptake and utilization of calcium and phosphorus [49]. AFB1 is known to have hepatotoxic and immunosuppressive effects in poultry, which may impair liver function and the immune system [3]. The liver is responsible for synthesizing vitamin D, essential for the absorption and metabolism of calcium and phosphorus, the main components of bone minerals [50]. AFB1 as a potent hepatotoxin directly damages liver cells by binding to DNA and causing mutations, triggering oxidative stress and disrupting essential cellular processes [3]. AFB1 has been shown to bind to the VDR, a protein that acts as the receptor for vitamin D [22]. This competitive binding effectively blocks vitamin D from interacting with the VDR, hindering its ability to maintain calcium and phosphorus homeostasis [23]. Chronic exposure to AFB1 can also lead to a decrease in the expression of VDR itself. This further reduces the number of available receptors for vitamin D, compounding the impairment of VDR signaling. Vitamin D deficiency is also linked with an impaired immune system which might further impair the bone remodeling process [51,52]. Therefore, AFB1 may affect the bone microstructure of broiler chickens by disrupting vitamin D synthesis, calcium and phosphorus homeostasis and the bone remodeling process. We also found that AFB1 up to 560 ppb does not affect intestinal permeability and the mRNA expression of tight junction proteins. Similar results were reported in previous studies, suggesting that AFB1 does not induce inflammation in the gastrointestinal tract [53]. AFB1 exhibits high intestinal absorption, meaning it is quickly taken up from the gastrointestinal tract (GIT) into the bloodstream [54,55]. This rapid uptake may limit its contact with the GIT and subsequent detrimental effects on the intestinal epithelium.

Recent studies have proposed a correlation between aflatoxicosis and the initiation of oxidative stress, inflammation and apoptosis, which might also affect bone remodeling [56,57]. Exposure to aflatoxins may trigger a series of cellular responses characterized by an increased production of reactive oxygen species, inflammatory reactions and programmed cell death [58]. These observations contribute to a growing body of evidence supporting the association between aflatoxin exposure and the induction of specific biological pathways, shedding light on potential mechanisms underlying the toxic effects of aflatoxicosis.

## 4. Conclusions

In conclusion, this study demonstrated that aflatoxin B1 at 230 ppb or higher administered for 19 days disrupted the cortical and trabecular structural formation of the long bones and reduced the cortical tissue volume and bone volume, indicating a reduction in total bone mass. At the molecular level, AFB1 at levels as low as 75 ppb impaired bone homeostasis via disruption of the vitamin D receptor and calcium and phosphorus transporters, potentially contributing to lameness and other bone disorders in broilers.

## 5. Materials and Methods

### 5.1. Preparation of Aflatoxin B1 Contaminated Feed

Aflatoxin B1 (AFB1), with a purity exceeding 98%, obtained from Cayman Chemical (Item No.: 11293, Ann Arbor, MI, USA), was utilized in the study. To create a stock solution, 10 mg of AFB1 was dissolved in 30 mL of methanol, following the procedure outlined by [59,60]. The resulting AFB1–methanol solution was thoroughly mixed with 5 kg of basal diet to achieve a 2 mg/kg concentration, forming a premix diet contaminated with AFB1. In parallel, a control diet premix was prepared by mixing 5 kg of the basal diet with an equivalent volume of methanol. Both the contaminated and control premixes were left overnight in a fume hood to facilitate the evaporation of methanol. The premixes were subsequently mixed with the basal diet at various levels to obtain five different levels of exposure: T1 (<2 ppb AFB1), T2 (75–79 ppb AFB1), T3 (150 ppb AFB1), T4 (230–260 ppb AFB1) and T5 (520–560 ppb AFB1). The reported levels of the AFB1 in the diets are based on the analyzed values of the aflatoxin in the finished feed. Strict safety measures, including protective eyewear, personal protective equipment and gloves, were used to prepare and handle the diets to minimize the risk of AFB1 exposure.

Finished feed samples were analyzed for aflatoxin B1 using the HPLC method at the Feed and Environmental Water Laboratory of the University of Georgia. Starter feed contained <2, 79, 150, 260 and 520 ppb of aflatoxin B1, while grower feed contained <2, 75, 150, 230 and 560 ppb of aflatoxin B1, respectively.

### 5.2. Birds and Experimental Design

A total of 360 one-day-old Cobb500 male broilers were randomly allocated to five treatment groups with six replicates containing 12 birds each. The birds were fed a corn-soybean-based diet with five different levels of aflatoxin B1 (AFB1) for 19 days. Treatment diets were prepared by mixing a basal diet with a premix contaminated with AFB1 to achieve the following target levels in the finished diets: T1 (<2 ppb AFB1), T2 (75–79 ppb AFB1), T3 (150 ppb AFB1), T4 (230–260 ppb AFB1) and T5 (520–560 ppb AFB1). Birds were fed each treatment diet in two phases: starter (0 to 8 days) and grower (9 to 19 days) (Table 3). Birds were raised in environmentally controlled battery cages with ad libitum access to feed and water throughout the experiment. Housing temperature was closely monitored and controlled, starting at 32 °C and gradually decreasing to 23 °C by Day 19. Lighting followed a standard schedule of 23 h light and 1 h dark for the first week, then transitioned to 18 h light and 6 h dark for the remaining 12 days. The birds were checked twice daily to ensure their well-being, and factors such as room temperature, bird condition, mortality and feed and water availability were thoroughly monitored during each inspection to ensure optimal growth and health conditions.

Throughout the study, mortality was recorded, and at the end of each diet phase on Days 8 and 19, body weight and feed intake were recorded at the replicate pen level (i.e., six replicate pens per treatment group). Subsequently, individual bird averages were derived by dividing pen totals by the number of birds, adjusted to the mortality. These individual values were then reported as whole numbers for body weight, body weight gain and feed intake and the hundredth for feed conversion ratio.

### 5.3. Intestinal Permeability

Intestinal permeability was measured using fluorescein isothiocyanate dextran (FITC-d; MW 4000; Sigma-Aldrich, St. Louis, MO, USA), modified by the method described by a previous study [61]. Briefly, on Day 19, one bird was randomly selected from each cage and orally inoculated with 1 mL of the FITC-d solution (2.2 mg/mL). Blood was collected from the birds two hours post-inoculation and allowed to clot for two hours in the dark at room temperature. After centrifugation at 1500× *g* for 15 min, serum was collected. A standard curve was generated using serial dilutions of the FITC-d stock, while a dilution buffer was created using pooled serum from birds on a basal diet. Standards and samples were loaded onto black 96-well plates (Greiner BIO-ONE, Monroe, NC, USA), and the FITC-d concentrations were quantified using a spectrophotometer (VICTOR Nivo Multimode Microplate Reader, PerkinElmer, Shelton, CT, USA) at an excitation wavelength of 485 nm and an emission wavelength of 528 nm.

### 5.4. RNA Extraction and Real-Time RT-PCR

RNA extraction, cDNA synthesis and real-time RT-PCR were performed to investigate the gene expression of the vitamin D receptor, calcium and phosphorus transporter genes and tight junction proteins in the jejunum. RNA was extracted using QIAzol lysis reagent (Qiagen, Valencia, CA, USA), according to the manufacturer’s instructions. The quantity and purity of the RNA were assessed using a NanoDrop™ Eight Spectrophotometer (Thermo Fisher Scientific, Waltham, MA, USA). Reverse transcription was carried out using the High-Capacity cDNA synthesis kit (Applied Biosystems, Foster City, CA, USA), and the obtained cDNA was diluted 10× prior to use in the PCR reaction. The primers used for gene expression analysis are shown in Table 4. Real-time RT-PCR was performed using SYBR Green Master Mix (Bio-Rad, Hercules, CA, USA) with a QuantStudio™ 3 Real-Time PCR System (Thermo Fisher Scientific, Waltham, MA, USA). The final PCR reaction volume was 10 μL, consisting of 5 μL of SYBR Green Master Mix, 2.5 μL of cDNA, 0.5 μL each of forward and reverse primers (10 μM) and 1.5 μL of water. The thermal cycle conditions were as follows: 95 °C denaturation for 5 min, 40 cycles at 95 °C for 15 s, 58 °C for 30 s and 72 °C for 30 s, followed by 95 °C for 15 s, 60 °C for 1 min and 95 °C for 15 s.

### 5.5. Bone Ash Analysis

The right tibia bones were collected on Days 8 and 19 from one bird per replicate (6 bones/treatment) and stored at −20 °C until bone ash analysis. Bone ash analysis was performed using the parameters reported by [62,63]. Briefly, the initial wet weight of each bone was recorded, followed by drying at 100 °C for 24 h, and dry bone weight was recorded. To obtain the fat-free dry weight, the fat was extracted from the bones with hexane (Fisher Scientific, MA, USA) in a Soxhlet apparatus for 48 h at 70 °C and oven-dried again at 100 °C for 24 h and weighed. The bones were ashed in a furnace at 600 °C overnight, and the ash weight was measured. The bone ash percentage was calculated by dividing the ash weight by the fat-free dry weight.

### 5.6. Micro-Computed Tomography (Micro-CT) of Femur Bone

**Figure 6:**
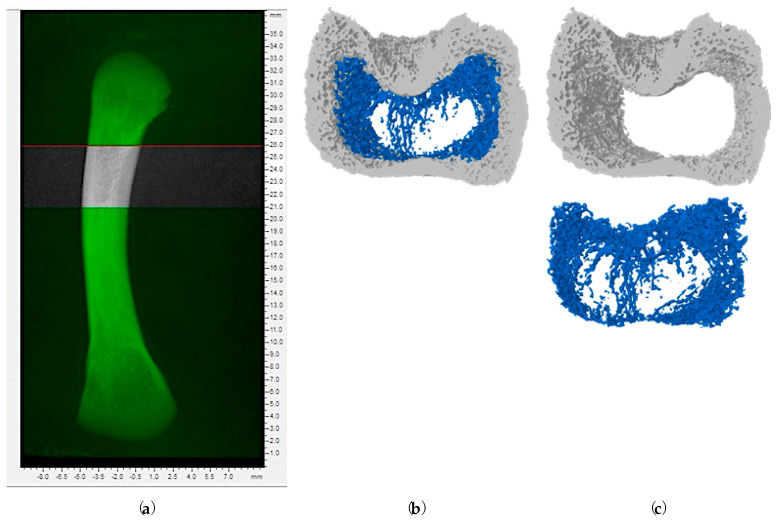
Selection of bone region for micro-computed tomography: (**a**) Selection of volume-of-interests (VOI), the VOI was chosen to begin from 50 slides (1.25 mm) below the nutrient foramen in the distal femur and to extend 200 slides (5 mm) for analysis. This specific region encompasses cortical bone and trabecular bone, making it an ideal area to represent bone quality; (**b**) Metaphyseal segment of bone chosen for separation and analysis; (**c**) Cortical (upper) and trabecular (lower) regions of bone segments separated from metaphyseal bone segment for further analysis.

The right femur bones were collected from one bird per replicate (6 bones/treatment) at 8 and 19 days of the experiment to evaluate bone morphological and microarchitectural changes using the parameters reported in a previous paper from our lab (Table 5) [64]. Prior to scanning, soft tissues surrounding the bones were removed, and bones were wrapped in a cheesecloth to prevent them from drying as described by [65]. The bone was held in a low-density 50 mL tube, and extra cheesecloth was used to keep the sample firmly inside the tube holder in a vertical orientation. The tube was then mounted on the scanning stage. Skyscan 1275—Micro-CT Scanner (Bruker Corporation, Kontich, Belgium) was used to scan the bones at a source voltage of 75 kV and a source current of 133 μA. Before scanning, an alignment test and flat field correction were performed following the guidelines outlined in the micro-CT manual (Bruker Corporation, Kontich, Belgium). Random movement and 180-degree scanning were utilized to decrease beam hardening. The dynamic range for all samples was established at 0–0.025. The volume of interest is illustrated in Figure 6. A customized process for bone separation was carried out on the 3D model. The separation process was based on the distinct density and morphology traits of each bone part. CTan (Version: 1.16.4.1; Bruker Corporation, Kontich, Belgium) was used to analyze the 3D model. A threshold of 85–255 (grayscale) was applied for all the bone samples. Two solid-state phantoms composed of calcium hydroxyapatite were utilized for calibration.

### 5.7. Statistical Analysis

The mean and pooled standard error of the mean were calculated for all experimental data. The data were tested for the normality of studentized residuals and homogeneity of variances. One-way ANOVA and Tukey HSD tests were performed using JMP Pro 17 (SAS Institute, Cary, NC, USA) to compare the treatment groups. Statistical significance was set at a *p*-value < 0.05, and a *p*-value between 0.05 and 0.1 indicated a tendency toward significance [66]. Linear and quadratic regressions using LSMean contrast in JMP Pro 17 were used to assess the effects of increasing AFB1 doses on each parameter.

## Figures and Tables

**Table 1:** Effect of increasing dosage of Aflatoxin B1 on growth performance of broilers during 0 to 8, 8 to 19 and 0 to 19 days of the experiment ^1^.

Items ^2^	Treatment Groups ^3^					SEM	*p*-Values ^4^		
	T1	T2	T3	T4	T5		P_Model_	P_Linear_	P_Quadratic_
0–8 days									
BW (g)	212	208	207	198	200	6	0.4675	0.0867	0.8741
BWG (g)	166	162	161	152	154	6	0.4640	0.0856	0.8772
FI (g)	197	198	195	195	190	5	0.8832	0.3644	0.6818
FCR (g/g)	1.19	1.22	1.21	1.32	1.24	0.06	0.5331	0.2490	0.5993
9–19 days									
BW (g)	705	707	699	693	688	16	0.9083	0.3473	0.8347
BWG (g)	493	499	492	495	488	15	0.9898	0.7618	0.7738
FI (g)	857	860	859	852	853	23	0.9990	0.8431	0.9025
FCR (g/g)	1.74	1.72	1.75	1.72	1.76	0.06	0.9846	0.8204	0.8157
0–19 days									
BWG (g)	659	661	653	647	641	16	0.9076	0.3462	0.8335
FI (g)	1053	1058	1053	1048	1044	24	0.9946	0.7007	0.8378
FCR (g/g)	1.60	1.60	1.62	1.62	1.63	0.05	0.9831	0.5514	0.9858

^1^ The data represent individual bird growth parameters obtained by averaging data from six replicate pens per treatment, with 12 birds per pen for 0–8 days and 9 birds per pen for 9–19 days adjusted with mortality. Values for 0–19 days were calculated by averaging across pens and treatments after individual bird parameters were derived. SEM represents the pooled standard error of the mean for each parameter across all treatments and time points. ^2^ Items: BW, body weight; BWG, body weight gain; FI, feed intake; FCR, feed conversion ratio. ^3^ Treatment groups: T1 (<2 ppb AFB1), T2 (75–79 ppb AFB1), T3 (150 ppb AFB1), T4 (230–260 ppb AFB1), T5 (520–560 ppb AFB1). ^4^ *p*-values: P_Model_, *p*-value for the whole model; P_Linear_, *p*-value for the linear regression; P_Quadratic_, *p*-value for the quadratic regression.

**Table 2:** Effect of increasing the dosage of an aflatoxin B1 contaminated diet on the microstructure of femoral metaphysis on Day 19 ^1^.

Bone Region	Parameters ^2^	Unit	Treatment Groups ^3^						*p*-Values ^4^		
			T1	T2	T3	T4	T5	SEM	PModel	PLinear	PQuadratic
Total	BMC	g	0.077 ^a^	0.071 ^ab^	0.069 ^ab^	0.062 ^b^	0.063 ^b^	0.003	0.0212	0.0015	0.523
	BMD	g/cm^3^	0.280	0.272	0.273	0.277	0.262	0.010	0.8026	0.3726	0.7833
	TV	mm^3^	275 ^a^	260 ^ab^	254 ^ab^	226 ^b^	240 ^ab^	10	0.0412	0.0063	0.3953
	BV	mm^3^	109 ^a^	99 ^ab^	96 ^ab^	85 ^b^	90 ^ab^	5	0.0406	0.0045	0.2972
	BV/TV	%	39.62	38.34	37.71	37.89	37.47	1.62	0.9011	0.3782	0.6894
	Po.V(cl)	mm^3^	0.245	0.217	0.204	0.129	0.146	0.030	0.0642	0.0069	0.8076
	Po.V(op)	mm^3^	165.7	160.0	158.4	140.5	150.1	8.5	0.3087	0.0781	0.665
	PP	%	60.38	61.66	62.29	62.11	62.53	1.62	0.9011	0.3782	0.6894
Cortical	BMC	g	0.057	0.051	0.051	0.045	0.048	0.003	0.0907	0.0175	0.2361
	BMD	g/cm^3^	0.475	0.491	0.495	0.515	0.517	0.010	0.0922	0.0075	0.7665
	TV	mm^3^	120 ^a^	105 ^ab^	102 ^ab^	89 ^b^	94 ^b^	6	0.0189	0.0020	0.2178
	BV	mm^3^	99 ^a^	89 ^ab^	87 ^ab^	76 ^b^	83 ^ab^	5	0.0429	0.0075	0.1968
	BV/TV	%	82.4 ^b^	85.0 ^ab^	85.0 ^ab^	86.3 ^ab^	88.1 ^a^	1.0	0.0337	0.0023	0.9663
	Po.V(cl)	mm^3^	0.237 ^a^	0.197 ^ab^	0.191 ^ab^	0.115 ^b^	0.139 ^ab^	0.029	0.0500	0.0060	0.5907
	Po.V(op)	mm^3^	21.06 ^a^	15.58 ^ab^	15.06 ^ab^	12.80 ^b^	10.93 ^b^	1.57	0.0067	0.0004	0.4195
	PP	%	17.58 ^a^	15.00 ^ab^	15.04 ^ab^	13.66 ^ab^	11.88 ^b^	1.04	0.0337	0.0023	0.9663
Trabecular	BMC	g	0.018 ^a^	0.017 ^ab^	0.016 ^ab^	0.015 ^ab^	0.012 ^b^	0.001	0.0331	0.0026	0.2792
	BMD	g/cm^3^	0.120 ^a^	0.116 ^ab^	0.114 ^ab^	0.113 ^ab^	0.090 ^b^	0.006	0.0214	0.0043	0.1336
	TV	mm^3^	146	146	144	129	138	8	0.5477	0.2219	0.8596
	BV	mm^3^	7.38	8.06	6.44	6.80	5.04	0.86	0.2328	0.0531	0.4096
	BV/TV	%	5.08	5.4	4.56	5.09	3.71	0.54	0.2607	0.0976	0.3414
	Tb.Th	mm	0.122	0.151	0.134	0.148	0.125	0.017	0.6706	0.954	0.2572
	Conn.Dn	mm^−3^	9.42	7.71	6.01	8.06	7.98	0.79	0.0809	0.3224	0.0259
	Tb.N	mm^−1^	0.438	0.348	0.289	0.370	0.371	0.041	0.1821	0.3946	0.0463

^a,b^ Means within a column not sharing a common letter differ significantly (*p* < 0.05). ^1^ The data represent the mean values of six femur bone samples per treatment. SEM represents the pooled standard error of the mean for each parameter across all treatment groups. ^2^ Parameters: BMC, bone mineral content; BMD, bone mineral density; TV, tissue volume; BV, bone volume; BV/TV, bone volume/tissue volume; Po.V(cl), volume of closed pores; Po.V(op), volume of open pores; Po(tot), total porosity percentage; Tb.Th, trabecular thickness; Conn.Dn, connectivity density; Tb.N, trabecular number. ^3^ Treatment groups: T1 (<2 ppb AFB1), T2 (75–79 ppb AFB1), T3 (150 ppb AFB1), T4 (230–260 ppb AFB1), T5 (520–560 ppb AFB1). ^4^ *p*-values: P_Model_, *p*-value for the whole model; P_Linear_, *p*-value for the linear regression; P_Quadratic_, *p*-value for the quadratic regression.

**Table 3:** Ingredient composition and calculated nutrient composition of the basal diet for starter and grower phases.

Ingredients, %	Starter	Grower
Corn	58.50	63.72
Soybean Meal (48% CP)	34.75	29.70
Soybean Oil	0.50	0.50
Dicalcium Phosphate	2.08	1.35
Limestone	1.02	1.06
DL-Methionine	0.33	0.32
L-Lysine HCl	0.24	0.27
L-Threonine	0.12	0.12
Common Salt	0.40	0.35
Vitamin Premix ^1^	0.10	0.10
Mineral Premix ^2^	0.08	0.08
Sand	1.88	2.13
Chromic Oxide	0.00	0.30
Total	100.00	100.00
Calculated Values, %		
D.M. ^3^	87.63	87.20
M.E., Kcal/g ^4^	2.90	2.95
Crude Protein	21.50	19.50
Calcium	0.96	0.80
Available Phosphorus	0.54	0.40
dLYS	1.26	1.16
dMET	0.65	0.61

^1^ Supplied per kilogram of diet: vitamin A, 3527 IU; vitamin D3, 1400 ICU; vitamin E, 19.4 IU; vitamin B12, 0.008 mg; Menadione, 1.1 mg; Riboflavin, 3.53 mg; d-Pantothenic Acid, 5.47 mg; Thiamine, 0.97 mg; Niacin, 20.28 mg; vitamin B6, 1.45 mg; Folic Acid, 0.57 mg; Biotin, 0.08 mg. ^2^ Supplied per kg of diet: Ca, 25.6 mg; Mn, 107.2 mg; Zn, 85.6 mg; Mg, 21.44 mg; Fe, 21.04 mg; Cu, 3.2 mg; I, 0.8 mg; Se, 0.32 mg. ^3^ D.M., dry matter of feed in percentage. ^4^ M.E., Kcal/g, metabolizable energy of feed in Kcal/g.

**Table 4:** Nucleotide sequences of the primers used for real-time RT-PCR.

Gene ^1^	Accession Number	Forward Primer	Reverse Primer
Housekeeping Genes			
GAPDH	NM_204305.2	GCTAAGGCTGTGGGGAAAGT	TCAGCAGCAGCCTTCACTAC
ACTB	NM_205518.2	CAACACAGTGCTGTCTGGTGGTA	ATCGTACTCCTGCTTGCTGATCC
Vitamin D Receptor			
VDR	NM_205098.2	GCAGCAGAAAGTCATCGACA	TGCTGAATTTGCTTCTCACG
Ca and P Transporters			
CALB1	NM_205513.2	AAGCAGATTGAAGACTCAAAGC	CTGGCCAGTTCAGTAAGCTC
CaSR	XM_416491.8	CTGCTTCGAGTGTGTGGACT	GATGCAGGATGTGTGGTTCT
NCX1	NM_001398209.1	TCACTGCAGTCGTGTTTGTG	AAGAAAACGTTCACGGCATT
NaPi-Ⅱb	NM_204474.3	AAAGTGACGTGGACCATG	GAGACCGATGGCAAGATCAG
PMCA1b	NM_001168002.4	TTAATGCCCGGAAAATTCAC	TCCACCAAACTGCACGATAA
Tight Junction Proteins			
MUC2	NM_001318434.1	ATGCGATGTTAACACAGGACTC	GTGGAGCACAGCAGACTTTG
CLDN1	NM_001013611.2	TGGAGGATGACCAGGTGAAGA	CGAGCCACTCTGTTGCCATA
ZO1	XM_015278981.2	CAACTGGTGTGGGTTTCTGAA	TCACTACCAGGAGCTGAGAGGTAA
OCLN	XM_025144248.1	GTCTGTGGGTTCCTCATCGT	GTTCTTCACCCACTCCTCCA

^1^ GAPDH, glyceraldehyde 3-phosphate dehydrogenase; ACTB, beta actin; CALB1, calbindin 1; CaSR, calcium-sensing receptor; NCX1, sodium-calcium exchanger 1; NaPi-IIb, sodium-phosphate cotransporter; PMCA1b, plasma membrane calcium ATPase 1b; VDR, vitamin D receptor, MUC2, mucin 2; CLDN1, claudin 1; ZO1, zonula occludens 1; OCLN, occludin.

**Table 5:** Definition of variables used for micro-CT.

Abbreviation	Variable	Description of Variables	Unit
BMC	Bone mineral content	Measure the bone mineral content of the tissue	g
BMD	Bone mineral density	Measure the bone mineral content per unit of volume	g/cm^3^
TV	Tissue volume	Volume of the entire region of interest	mm^3^
BV	Bone volume	Volume of the bone segment	mm^3^
BV/TV	Bone volume fraction	Bone volume segment volume as a fraction of tissue volume from the region of interest	%
Po.V(cl)	Volume of closed pores	Volume of closed pore space	mm^3^
Po.V(op)	Volume of open pores	Volume of open pore space	mm^3^
PP	Porosity percentage	The volume of pores by total volume of bone	%
Tb.Th	Trabecular thickness	Mean thickness of trabeculae measured using 3-D methods	mm
Conn.Dn	Connectivity density	A measure of the degree of connectivity of trabeculae normalized by TV	mm^−3^
Tb.N	Trabecular number	Average number of trabeculae per unit of length	mm^−1^

## Data Availability

The data presented in this study are available on request from the corresponding author.

## Supplementary Materials

The following supporting information can be downloaded at https://www.mdpi.com/article/10.3390/toxins16020078/s1, Supplementary Table S1. Effect of increasing the dosage of an aflatoxin B1 contaminated diet on the microstructure of femoral metaphysis on Day 8; Supplementary Table S2. Effect of increasing the dosage of an aflatoxin B1 contaminated diet on tibial bone parameters on Day 8; Supplementary Table S3. Effect of increasing the dosage of an aflatoxin B1 contaminated diet on mRNA levels of calcium and phosphorus transporter genes on Day 8; Supplementary Table S4. Effect of increasing the dosage of an aflatoxin B1 contaminated diet on mRNA levels of tight junction proteins on Day 8.
