# Supplementary material for: Aflatoxin B1 Impairs Bone Mineralization in Broiler Chickens
Source: Toxins (Basel). 2024 Feb 2;16(2):78. doi: 10.3390/toxins16020078 (PMC10893327; doi:10.3390/toxins16020078)
Supplement: Supplementary file 1 [file toxins-16-00078-s001.zip › toxins-2821307-supplementary.pdf]

Supplementary Materials: Aflatoxin B1 Impairs Bone Mineralization in Broiler Chickens

Supplementary Table S1. Effect of increasing dosage of Aflatoxin B1 contaminated diet on microstructure of femoral metaphysis on day 8 [1].

Bone Region	Parameters[2]	Unit	Treatment Groups[3]					SEM	P-values[4]		
			T1	T2	T3	T4	T5		P_{Model}	P_{Linear}	$P_{Quadratic}$
**Total**	**BMC**	g	0.016	0.019	0.018	0.018	0.020	0.001	0.5944	0.2700	0.81380
	**BMD**	g/mm[3]	0.28	0.33	0.32	0.31	0.34	0.02	0.1163	0.0907	0.4325
	**TV**	mm[3]	58.2	56.2	56.5	56.7	57.9	2.8	0.9817	0.9934	0.5577
	**BV**	mm[3]	25.8	29.9	29.0	27.4	31.3	2.5	0.5777	0.2999	0.8889
	**BV/TV**	-	44.2	52.6	51.2	47.7	53.9	2.7	0.1104	0.1042	0.5284
	**TS**	mm[2]	94	92	94	93	94	3	0.9887	0.9255	0.6830
	**BS**	mm[2]	165	155	159	163	153	10	0.8961	0.6146	0.9804
	**BS/BV**	mm[-1]	6.6	5.4	5.6	6.1	5.0	0.4	0.0619	0.0484	0.6849
	**BS/TV**	mm[-1]	2.84	2.77	2.81	2.86	2.65	0.09	0.4817	0.3110	0.4111
**Cortical**	**BMC**	g	0.014	0.017	0.016	0.015	0.017	0.001	0.3981	0.2452	0.6860
	**BMD**	g/mm[3]	0.506	0.544	0.531	0.530	0.538	0.013	0.3768	0.2750	0.3784
	**TV**	mm[3]	26.6	30.9	30.2	28.2	32.1	2.6	0.6387	0.3439	0.8409
	**BV**	mm[3]	25.2	29.5	28.7	26.8	30.9	2.4	0.5313	0.2820	0.8853
	**BV/TV**	-	94.8	95.8	95.1	95.3	96.5	0.7	0.5683	0.2634	0.6404
	**TS**	mm[2]	135.2	129.3	131.2	132.4	131.3	3.8	0.8718	0.7110	0.5491
	**BS**	mm[2]	154.7	147.5	152.5	153.4	147.4	8.5	0.9614	0.7562	0.9571
	**BS/BV**	mm[-1]	6.36	5.19	5.40	5.84	4.82	0.38	0.0850	0.0616	0.7310
	**BS/TV**	mm[-1]	6.03	4.97	5.13	5.57	4.64	0.35	0.0954	0.0700	0.6872
**Trabecular**	**BMC**	g	0.0021	0.0018	0.0017	0.0020	0.0017	0.0001	0.3461	0.3300	0.4285
	**BMD**	g/mm[3]	0.0736	0.0810	0.0745	0.0770	0.0761	0.0036	0.6877	0.9246	0.5888
	**TV**	mm[3]	28.12	21.96	23.17	25.20	22.76	1.42	0.0621	0.1334	0.1597

**BV**	mm³	0.222	0.181	0.144	0.244	0.128	0.048	0.4560	0.4510	0.9460
**BV/TV**	-	0.786	0.847	0.623	0.927	0.509	0.181	0.5473	0.4436	0.5545
**TS**	mm²	63.2	53.6	55.5	58.6	54.4	2.1	0.0373	0.0898	0.1657
**BS**	mm²	10.5	7.4	6.6	10.0	5.6	1.7	0.2382	0.2152	0.8169
**BS/BV**	mm⁻¹	50.0	45.7	46.7	44.7	48.3	3.4	0.8270	0.6861	0.3298
**BS/TV**	mm⁻¹	0.379	0.346	0.287	0.385	0.227	0.061	0.3690	0.2021	0.6971

[1] Data represents mean values of six femur bone samples per treatment. SEM represents the pooled standard error of the mean for each parameter across all treatment groups.

[2] Parameters: BMC, bone mineral content; BMD, bone mineral density; TV, tissue volume; BV, bone volume; BV/TV, bone volume/ tissue volume; TS, tissue surface area; BS, bone surface area; BS/BV, bone surface area/ bone volume; BS/ TV, bone surface area/ tissue volume.

[3] Treatment groups: T1 (< 2 ppb AFB1), T2 (75-79 ppb AFB1), T3 (150 ppb AFB1), T4 (230-260 ppb AFB1), T5 (520-560 ppb AFB1).

[4] P-values: P_{Model}, p-value for the whole model; P_{Linear}, p-value for the linear regression; $P_{Quadratic}$, p-value for the quadratic regression

Supplementary Table S2. Effect of increasing dosage of Aflatoxin B1 contaminated diet on tibial bone parameters on day 8 [1].

Parameters[2]	Treatment Groups[3]					SEM	P-values[4]		
	T1	T2	T3	T4	T5		P_{Model}	P_{Linear}	$P_{Quadratic}$
FBW (g)	0.983	0.982	1.031	1.018	1.053	0.055	0.8879	0.3466	0.96515
DBW (g)	0.327	0.329	0.339	0.342	0.353	0.018	0.8516	0.2704	0.8540
FFDW (g)	0.322	0.330	0.335	0.333	0.356	0.020	0.8419	0.3077	0.7721
AW (g)	0.1454	0.1525	0.1548	0.1512	0.1656	0.0108	0.7745	0.2750	0.8342
Ash %	44.92	46.11	46.06	45.38	46.47	0.64	0.5247	0.2892	0.7509

[1] Data represents mean values of six tibia bone samples per treatment. SEM represents the pooled standard error of the mean for each parameter across all treatment groups.

[2] Parameters: FBW, fresh bone weight; DBW, dry bone weight; FFDW, fat free dry weight; AW, ash weight.

[3] Treatment groups: T1 (< 2 ppb AFB1), T2 (75-79 ppb AFB1), T3 (150 ppb AFB1), T4 (230-260 ppb AFB1), T5 (520-560 ppb AFB1).

[4] P_{Model}, p-value for the whole model; P_{Linear}, p-value for the linear regression; $P_{Quadratic}$, p-value for the quadratic regression.

Supplementary Table S3. Effect of increasing dosage of Aflatoxin B1 contaminated diet on mRNA levels of calcium and phosphorus transporter genes on day 8 [1].

**Genes**[1]	**Treatment Groups**[3]					**SEM**	**P-values**[4]		
	**T1**	**T2**	**T3**	**T4**	**T5**		P_{Model}	P_{Linear}	$P_{Quadratic}$
CALB1	1.00	1.07	1.80	1.62	0.87	0.39	0.4116	0.8166	0.1109
CASR	1.00	0.71	0.75	0.68	0.63	0.22	0.7679	0.2665	0.6418
NCX1	1.00	0.66	0.72	0.76	1.02	0.23	0.6989	0.8495	0.1713
NaPi-IIb	1.00	1.25	1.20	1.53	1.10	0.21	0.4980	0.4868	0.2370
PMCA1	1.00[ab]	1.40[a]	0.94[ab]	0.48[ab]	0.33[b]	0.25	0.0395	0.0086	0.2407
VDR	1.00	1.89	2.16	1.41	2.26	0.38	0.1530	0.1044	0.4585

[a-b] Means within a column not sharing a common letter differ significantly ($P < 0.05$).

[1] Data are presented as mean values of fold change in mRNA expression of calcium and phosphorus transporter genes using $2^{-\Delta\Delta Ct}$ method. Data relates to 6 jejunum samples per treatment.

[2] Genes: CALB1, calbindin 1; CaSR, calcium-sensing receptor; NCX1, sodium-calcium exchanger 1; NaPi-IIB, sodium-phosphate cotransporter; PMCA1b, plasma membrane calcium ATPase 1b; VDR, vitamin D receptor.

[3] Treatment groups: T1 (< 2 ppb AFB1), T2 (75-79 ppb AFB1), T3 (150 ppb AFB1), T4 (230-260 ppb AFB1), T5 (520-560 ppb AFB1).

[4] P_{Model}, p-value for the whole model; P_{Linear}, p-value for the linear regression; $P_{Quadratic}$, p-value for the quadratic regression.

Supplementary Table S4. Effect of increasing dosage of Aflatoxin B1 contaminated diet on mRNA levels of tight junction proteins on day 8 [1].

Genes[2]	Treatment Groups[3]					SEM	P-values[4]		
	T1	T2	T3	T4	T5		P_{Model}	P_{Linear}	$P_{Quadratic}$
MUC2	1.00	0.91	1.14	0.80	1.28	0.20	0.6294	0.5558	0.5192
CLDN1	1.00	0.48	1.21	0.57	0.95	0.26	0.3939	0.9944	0.6982
ZO1	1.00	0.72	0.85	0.57	0.75	0.12	0.1842	0.1092	0.284
OCLN	1.00	1.00	1.09	1.06	0.99	0.11	0.9715	0.9103	0.5989

[1] Data are presented as mean values of fold change in mRNA expression of tight junction related genes using $2^{-\Delta\Delta Ct}$ method. Data relates to 6 jejunum samples per treatment.

[2] Genes: MUC2, mucin 2; CLDN1, claudin 1; ZO1, zonula occludens; and OCLN, occluding.

[3] Treatment groups: T1 (< 2 ppb AFB1), T2 (75-79 ppb AFB1), T3 (150 ppb AFB1), T4 (230-260 ppb AFB1), T5 (520-560 ppb AFB1).

[4] P_{Model}, p-value for the whole model; P_{Linear}, p-value for the linear regression; $P_{Quadratic}$, p-value for the quadratic regression.
